# Selective targeting and modulation of plaque associated microglia via systemic hydroxyl dendrimer administration in an Alzheimer’s disease mouse model

**DOI:** 10.1186/s13195-024-01470-3

**Published:** 2024-05-06

**Authors:** Caden M. Henningfield, Neelakshi Soni, Ryan W. Lee, Rishi Sharma, Jeffrey L. Cleland, Kim N. Green

**Affiliations:** 1 Department of Neurobiology and Behavior, University of California, 3208 Biological Sciences III, Irvine, CA 92697 USA; 2 Ashvattha Therapeutics, Inc, Redwood City, CA 94065 USA

**Keywords:** Microglia, Alzheimer’s disease, Inflammation, Dendrimers, Amyloid

## Abstract

**Background:**

In Alzheimer’s disease (AD), microglia surround extracellular plaques and mount a sustained inflammatory response, contributing to the pathogenesis of the disease. Identifying approaches to specifically target plaque-associated microglia (PAMs) without interfering in the homeostatic functions of non-plaque associated microglia would afford a powerful tool and potential therapeutic avenue.

**Methods:**

Here, we demonstrated that a systemically administered nanomedicine, hydroxyl dendrimers (HDs), can cross the blood brain barrier and are preferentially taken up by PAMs in a mouse model of AD. As proof of principle, to demonstrate biological effects in PAM function, we treated the 5xFAD mouse model of amyloidosis for 4 weeks via systemic administration (ip, 2x weekly) of HDs conjugated to a colony stimulating factor-1 receptor (CSF1R) inhibitor (D-45113).

**Results:**

Treatment resulted in significant reductions in amyloid-beta (Aβ) and a stark reduction in the number of microglia and microglia-plaque association in the subiculum and somatosensory cortex, as well as a downregulation in microglial, inflammatory, and synaptic gene expression compared to vehicle treated 5xFAD mice.

**Conclusions:**

This study demonstrates that systemic administration of a dendranib may be utilized to target and modulate PAMs.

## Main points

1. Systemically injected hydroxyl dendrimers (HDs) can cross the Blood-brain-barrier (BBB) and are preferentially phagocytosed by plaque associated microglia (PAMs) in the brain of 5xFAD mice.
2. 5xFAD mice treated with HDs conjugated to a CSF1R inhibitor (D-45113) show significant reductions in amyloid pathology, microglial number, and inflammatory gene transcription.
3. HDs have therapeutic potential to cross a slightly impaired BBB and specifically target and modulate PAMs in AD while leaving other cell types unaffected.

## Introduction

Alzheimer’s disease (AD) is a progressive, age-related neurodegenerative disorder that is triggered by the appearance and build-up of amyloid-beta (Aβ) plaques in the cortex. Our lab and others have shown that microglia play an integral role in plaque formation and homeostasis, as well as downstream pathogenesis such as loss of synapses, perineuronal nets, and neurons [9]; Hansen, Hanson, & Sheng [20]; E. Spangenberg et al [61, 62].​ In addition to initiating the inflammatory response to disease pathology, phenotypically distinct microglia cluster around Aβ plaques and actively regulate plaque morphology (e.g., compaction) [6]; Condello, Yuan, Schain, & Grutzendler [8]. Moreover, recent genome-wide association studies associated single nucleotide polymorphisms of genes highly enriched or exclusively expressed in myeloid cells (including *Trem2*, *Tyrobp*, *Apoe*, *Ms4a*, *Abca7*, *Abi3*, *Spi1*) with an altered risk of developing AD [20, 24, 25, 32, 64]. These data indicate microglia as a mediator of AD and a potential therapeutic target.

Microglia surrounding plaques undergo significant physical and chemical changes, including the retraction of their processes and swelling of their cell bodies. These changes are mediated by extensive alterations in gene expression, which reprogram the microglia to mount inflammatory responses and remodel their metabolism and lipid handling [20]. As a result, they transition to a disease-associated microglia (DAM) phenotype, characterized by specific functional and molecular features. These changes in gene expression have been well studied by single cell RNA sequencing and find heterogenous subsets of microglia in AD, demarcated by the expression of genes such as *Trem2* and *Tyrobp* that conventionally differentiate between disease- vs. non-disease-associated microglia, which in AD roughly correspond to plaque- and non-plaque-associated microglia (PAM and NPAM) respectively [30]. Currently, the role of PAMs in AD is unclear as brain wide microglial gene deletion and overexpression studies have shown contradicting results [18]; Gratuze, Leyns, & Holtzman [17, 26, 27, 33, 34, 54, 68]. Thus, there is a critical need to specifically target and modulate PAMs over NPAMs to determine this cell population’s contribution to AD.

Dendranib precision nanomedicine is based on hydroxyl dendrimer (HD) technology. HDs consist of a hydrophobic core, repeating branches that expand outward, and hydrophilic functional groups at the outer surface. Importantly, the high density of surface hydroxyls provides a neutral charge allowing HDs to easily cross the blood brain barrier (BBB) in regions of inflammation and be selectively internalized by activated microglia and macrophages [44, 45, 47–49, 66].

Size and surface chemistry of dendrimers determine their toxicity and biodistribution [4]. More than 100 dendritic structures have been reported. Some dendrimers have been used clinically for nucleic acid and drug delivery in cancer, including many types of brain tumors [3, 29, 31, 42, 43, 71] and show potential application in gene therapy [1, 19, 40]. Notably, polyamidoamine (PAMAM) dendrimers have been shown to cross the BBB during times when pathological insults such as stroke, tumors, or traumatic brain injury compromise the BBB [56]. Traditional dendrimers alone do not bypass the BBB​ with high efficiency without resorting to invasive approaches such as carotid artery injections [63, 74]. Recently, however, systemic administration of HDs have shown promise in bypassing slightly impaired BBB and were shown to be taken up specifically by microglia and macrophages in regions of neuroinflammation in rodent models of cerebral palsy, glioblastoma, Rett syndrome, AD, ALS, and multiple sclerosis (MS) [44, 45, 47–49, 59, 60, 66]. In the AD brain, PAMs are the primary phagocytic macrophages; therefore, HDs theoretically have the capacity to bypass the BBB and become specifically engulfed by PAMs.

Here, we sought to determine whether HDs can successfully bypass the BBB and be specifically phagocytosed by PAMs in the context of AD.​ To that end, we intraperitoneally injected HDs conjugated to a Cy5 fluorophore into an aggressive mouse model of amyloidosis; 5xFAD mice at 7 months of age. We find that one injection is sufficient for the dendrimers to cross the BBB and leads to brain-wide, PAM-specific engulfment of these HDs into the microglial lysosomal compartment. To therapeutically modulates PAM function, as proof of principle, we used D-45113, a dendranib that inhibits CSF1R tyrosine kinase. Our lab has previously shown that all microglia express CSF1R, and that inhibition of CSF1R leads to the indiscriminate death of the microglia [12], and that the elimination of microglia in 5xFAD mice can inhibit plaque development early in disease, and rescue synapse and neuronal number associated with late disease [61, 62]. Additionally, our lab and others have shown that low-dose inhibition of CSF1R can inhibit microglia-plaque association, attenuate neuroinflammation and rescue synaptic integrity and cognition in AD mouse models [10, 51]. Not to be overlooked, studies inhibiting CSF1R in tauopathy models show reduced levels of microglia which leads to reductions in tau levels, amelioration of inflammation, and synaptic, and neuronal loss [2, 28, 41]. Altogether, HDs represent a novel and nuanced approach for targeting PAMs and further studies should be undertaken with other microglial modulators to uncover the specific role of PAMs in AD. Establishing the effectiveness of these dendrimers in targeting and treating PAMs will allow us to tailor appropriate therapies towards this subset of microglia and develop therapeutic treatments with greater precision.

## Methods

### Synthesis of D-45113

D4-alkyne dendrimer (Lot# DP-07-85-3) was dissolved in 20 mL of anhydrous dimethylacetamide (DMA). A CSF1R tyrosine kinase inhibitor with a terminal azide was added to a stirring solution of D4-alkyne. Copper bromide and Pentamethyldiethylenetriamine (PMDTA) were then added to the solution. The stirring solution was placed in a 95ºC oil bath overnight. The reaction mixture was then dialyzed against DMA followed by water (membrane cut-off at 1000 Da). The aqueous solution was then lyophilized to obtain D-45113.

### Mice

All animal experiments performed in this study were approved by the UC Irvine Institutional Animal Care and Use Committee (IACUC) and were compliant with ethical regulations for animal research and testing. Mice were mixed sex C57BL/6 (000664) mice. Animals were housed with open access to food and water under 12 h/12 h light-dark cycles. All mice were aged to 5 or 12 months unless otherwise indicated. The 5xFAD mouse expresses five familial AD genes (APP Swedish, Florida, and London; PSEN1 M146L + L286V; [50] and is characterized by aggressive amyloid pathology throughout the brain and synaptic and neuronal loss in the subiculum. For 5xFAD genotyping, the primer sequences used were PS1 Forward 5′ - AAT AGA GAA CGG CAG GAG CA – 3′ and PS1 Reverse 5′ - GCC ATG AGG GCA CTA ATC AT – 3′.

### Animal treatments

All rodent experiments were performed in accordance with animal protocols approved by the Institutional Animal Care and Use Committee (IACUC) at the University of California, Irvine. 7-month-old wild-type (WT) or 5xFAD mice were intraperitoneally (IP) injected with 55 mg/kg G4 PAMAM hydroxyl dendrimers conjugated to a Cy5 fluorophore followed by euthanasia 48 h post injection. For time course D-Cy5 experiments, 7–9-month-old mice were treated as above, but euthanized either 48 h, 15 days, or 21 days post injection. For D-45113 experiments, 4 month and 11-month-old mice were IP injected with 200 mg/kg of D-45113 twice per week for four weeks. At the end of treatments, mice were euthanized via CO_2_ inhalation and transcardially perfused with 1X phosphate buffered saline (PBS). For all studies, brains were removed, and hemispheres separated along the midline. Brain halves were either flash frozen for subsequent biochemical analysis, or drop-fixed in 4% Paraformaldehyde (PFA; Thermo Fisher Scientific, Waltham, USA) for subsequent immunohistochemical analysis. Half brains collected into 4% PFA for 48 h and then transferred to a 30% sucrose solution with 0.02% sodium azide for another 48–72 h at 4 C. Fixed half brains were sliced at 40 μm using a Leica SM2000 R freezing microtome.

### Histology and confocal microscopy

Fluorescent immunolabeling was performed using a standard indirect technique as described previously [22]. Brain sections were stained with primary antibodies against: ionized calcium binding adaptor molecule 1 (IBA1; 1:1000; 019-19741, Wako and ab5076, Abcam), CD68 (1:200; BioRad) glial fibrillary protein (GFAP; 1:1000; Abcam), NeuN (1:1000; Millipore), OLIG2 (1:200; Abcam), Aβ1–16 (6E10; 1:1000; Biolegend), and anti-lysosomal associated membrane protein 1 (LAMP1; 1:200; Santa Cruz Biotechnologies). For Amylo-Glo staining (TR-300-AG; Biosensis), tissue sections were washed in 70% ethanol 1 × 5 min, followed by a 1 × 2 min wash in distilled water. Sections were then placed in a 1% Amylo-Glo solution for 1 × 10 min then washed with 0.9% saline for 1 × 5 min and distilled water for 1 × 15 s before continuing fluorescent immunolabelling. For Thioflavin-S (Thio-S) staining, tissue sections were placed for 1 × 10 min incubation in 0.5% Thio-S (1892; Sigma-Aldrich) diluted in 50% ethanol. Sections were then washed 2 × 5 min each in 50% ethanol and one 10-min wash in 1xPBS before continuing with fluorescent immunolabelling.

High resolution fluorescent images were obtained using a Leica TCS SPE-II confocal microscope and LAS-X software. For confocal imaging, one field of view (FOV) per brain region was captured per mouse unless otherwise indicated.

### Aβ and NfL ELISA

To isolate protein for the ELISA, flash-frozen brain hemispheres were microdissected into cortical, hippocampal, and thalamic regions and grounded to a powder. Hippocampal tissue was then homogenized in Tissue Protein Extraction Reagent (TPER (Life Technologies, Grand Island, NY)) with protease and phosphatase inhibitors present. Samples were centrifuged at 100,000 g for 1 h at 4 °C to generate TPER-soluble fractions. To generate formic acid fractions, protein pellets from the TPER-soluble fraction were then homogenized in 70% formic acid and centrifuged at 100,000 g for 1 h at 4 °C, the formic acid fraction is then neutralized. Quantification of soluble and insoluble fractions of both Aβ and NfL was performed as previously described [67].

### RNA sequencing

Whole transcriptome RNA sequencing (RNA-Seq) libraries were produced from hippocampal tissue of WT/Veh, WT/D-45113, 5xFAD/Veh, and 5xFAD/D-45113 mice sacrificed at 12 months of age. RNA was isolated with an RNA Plus Universal Mini Kit (Qiagen, Valencia, USA) according to the manufacturer’s instructions. Library preparation, RNA-seq, and read mapping analysis were performed by Novogene Co. Gene expression was analyzed using Limma, edgeR, and org.Mm.eg.db packages (Robinson, McCarthy, & Smyth [55]), with expression values normalized into FPKM (fragments per kilobase of transcript per million mapped reads). Differentially-expressed genes were selected by using false discovery rate (FDR) < 0.05. Heatmaps were created using Morpheus (Morpheus, https://software.broadinstitute.org/morpheus) and volcano plots were created using VolcaNoseR [16].

### Data analysis and statistics

Both male and female mice were used in all statistical analyses. ThioS, IBA1, NeuN, and OLIG2 counts were measured via the spots function and 6E10, LAMP1, and GFAP volume were measured via the surfaces function on Imaris version 9.6. All analyses were performed on 20x images (550 μm X 550 μm). The number of dendrimer^+^ cells / FOV in the subiculum and somatosensory cortex were manually counted for 20x images (550 μm X 550 μm) for each mouse via ImageJ. Number of PAMs and NPAMs with dendrimer present in their lysosome were then counted and divided by the total number of PAMs and NPAMs, respectively to get the ratio of PAMs and NPAMs containing Cy5 dendrimer.

Statistical analysis was performed with Prism Graph Pad (v.8.0.1; La Jolla, USA). To compare two groups, the unpaired or paired Student’s t-test was used. Time-course data was analyzed using One-way ANOVA (48 h, 15 days, and 21 days), while D-45113 data with more than two groups used Two-way ANOVA (Treatment: Vehicle vs. D-45113 and Genotype: WT vs. 5xFAD) using GraphPad Prism Version 8. Tukey’s post hoc tests were employed to examine biologically relevant interactions from the two-way ANOVA regardless of statistical significance of the interaction. For all analyses, statistical significance was accepted at *p* < 0.05. and significance expressed as follows: *p < 0.05, **p < 0.01, ***p < 0.001. n is given as the number of mice within each group. Statistical trends are accepted at *p* < 0.10 (^#^). Data are presented as raw means and standard error of the mean (SEM).

## Results

### Hydroxyl dendrimers are phagocytosed exclusively by PAMs in the 5xFAD mouse brain

**Fig. 1:**
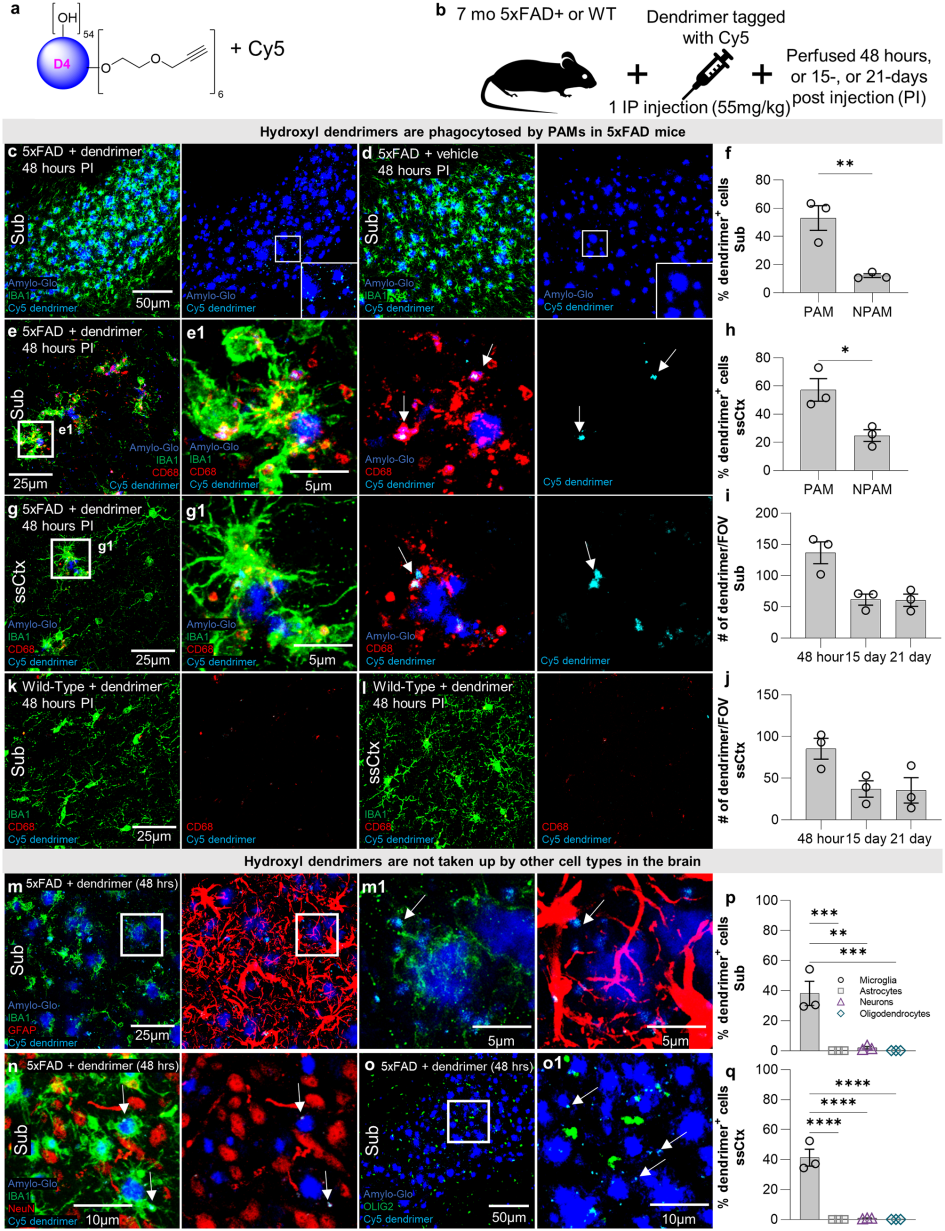
G4 HDs are phagocytosed by plaque associated microglia (PAM). Schematic HD conjugated to a Cy5 fluorophore (D-Cy5) (**a**). Experimental paradigm: 5xFAD mice were IP injected with D-Cy5 and perfused either 48 h, 15 days, or 21 days post injection (**b**). Representative 20x confocal microscopy images of the subiculum at 48 h post injection (**c**) show D-Cy5 localization in plaque-heavy areas, while 5xFAD mice injected with vehicle do not show Cy5 signal (**d**). 63x (**e**, **g**) and inset images (**e1**, **g1**) of the hippocampus (**e**) and somatosensory cortex (**g**) highlight D-Cy5 colocalization within microglial lysosomes (CD68). Arrows indicate areas where dendrimers colocalize with CD68. Quantification of 20x images of the percentage of plaque-associated microglia (PAM) and non-plaque-associated microglia (NPAM) containing dendrimer reveal that D-Cy5 are taken up primarily by PAM and to a lesser extent by NPAM in the subiculum (**f**) and somatosensory cortex (**h**) 48 h post injection. Quantification of the number of D-Cy5 puncta present in a 20x FOV at 48 h, 15 days, and 21 days post injection in the hippocampus and somatosensory cortex indicate that HDs persist in the brain through 21 days in the subiculum (**i**) and somatosensory cortex (**j**). Representative 20x images of WT mice injected with dendrimer show small amounts of dendrimer present in the subiculum (**k**) and somatosensory cortex (**l**) 48 h post injection. 63x representative images (**m**) and zoomed in images (**m1**) of GFAP (astrocytes) and IBA1 staining, 63x images of IBA1 and NeuN (neurons) staining (**n**), and 20x (**o**) and zoomed in images of OLIG2 (oligodendrocytes) staining show virtually no uptake of D-Cy5 in any of these cell types in the subiculum (**p**) nor somatosensory cortex (**q**). Statistical analysis for (**f**, **h**) used a two-tailed t-test; (**i**, **j**, **p**, **p**) used a one-way ANOVA with Tukey’s multiple comparison test. Significance indicated as * *p* < 0.05; ** *p* < 0.01; *** *p* < 0.001

To determine whether HDs can be used as a pharmacological tool to target PAMs in an AD mouse model, we utilized HDs conjugated to a Cy5 fluorophore (referred to as D-Cy5) as previously described [44, 45, 47–49, 66] (Fig. 1a). 7-month-old 5xFAD mice were intraperitoneally injected with D-Cy5 or saline and subsequently perfused 48 h, 15 days, or 21 days post injection (Fig. 1b). Cy5 signal is seen in the brain of 5xFAD, with a marked abundance of Cy5 around dense core plaques stained with Amylo-Glo (Fig. 1c), while control 5xFAD mice show no Cy5 signal in the brain (Fig. 1d). Upon closer inspection, D-Cy5 colocalizes with microglia (IBA1) and microglial lysosomes (CD68), in both the hippocampus (Fig. 1e, e1) and somatosensory cortex (Fig. 1g, g1), clustered around plaques, indicating that PAMs may phagocytose D-Cy5. Importantly, wild-type (WT) mice treated with D-Cy5 show very little to no Cy5 signal in the hippocampus (Fig. 1k) and somatosensory cortex (Fig. 1l); however, when signal is present, D-Cy5 colocalizes with microglia and their lysosomes. Quantification shows that 55% and 60% of PAMs as well as 20% and 15% of NPAMs contain D-Cy5 in the hippocampus and somatosensory cortex, respectively in 5xFAD mice (Fig. 1f and h), showing a preferential targeting of PAMs vs. NPAMs. Further, D-Cy5 is present in 5xFAD brains at 48 h, 15 days, and persists up to 21 days post injection in both the subiculum (Fig. 1j) and somatosensory cortex (Fig. 1j). To determine if any other cell types in the 5xFAD brain take up D-Cy5 we stained for astrocytes (GFAP; Fig. 1m, m1), neurons (NeuN; Fig. 1n), and oligodendrocytes (OLIG2; Fig. 1o, o1) and find virtually no D-cy5 signal in any of these cell types in the subiculum (Fig. 1p) nor somatosensory cortex (Fig. 1q).

### D-45113, a dendranib that inhibits CSF1R, rescues Elevated Plus Maze (EPM) performance and Aß burden in 12-month-old 5xFAD mice

**Fig. 2:**
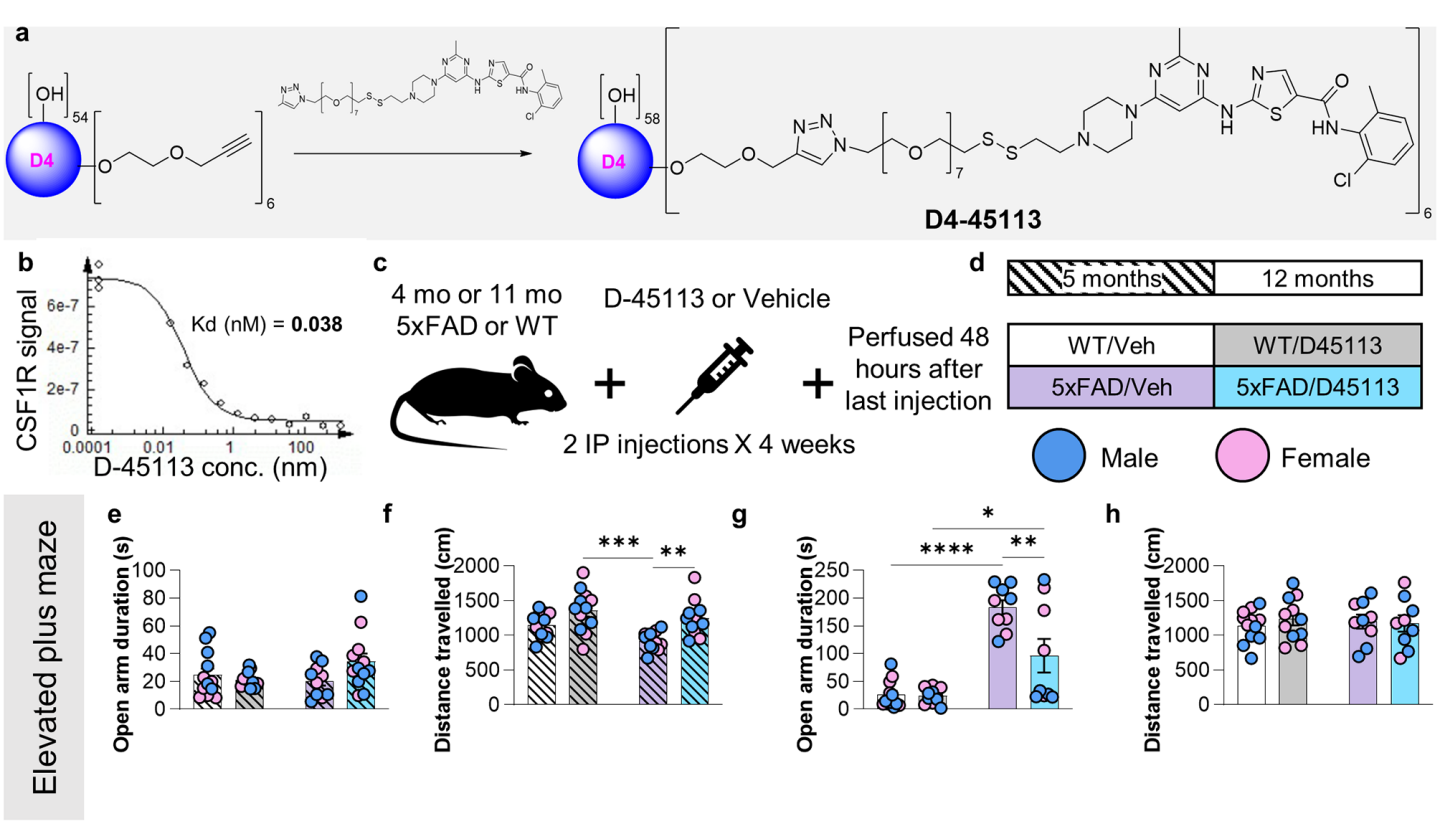
Intraperitoneal administration D-45113 rescues elevated plus maze performance in 5xFAD mice. Schematic of D-45113, a dendranib that inhibits CSF1R tyrosine kinase (**a**). Graph measuring the amount of CSF1R kinase plotted against D-45113 concentration (Kd = 0.04) (**b**). Experimental paradigm schematic revealing that 4- and 11-month-old 5xFAD and wild-type (WT) mice were injected with D-45113 or a vehicle for a total of 8 injections over the course of a month, at which point animals were perfused at either 5- or 12-months of age (**c**). All subsequent analysis and images of the 5-month timepoint are indicated by diagonal hatch lines while the 12-month timepoint is solidly filled, with male data-points filled with blue, and female data-points filled with pink (**d**). Elevated plus maze data at the 5-month timepoint indicate no changes are seen in the amount of time spent in the open maze arms with dendrimer treatment nor with 5xFAD genotype (**e**), however; WT/D-45113 and 5xFAD/D-45113 mice traveled a greater distance over the course of the 5-minute trial compared to 5xFAD/Veh mice (**f**). In the 12-month timepoint, the 5xFAD groups both showed a significant increase in the duration of time spent in the open arms of the maze, as expected, but strikingly, 5xFAD/D-45113 mice showed a significant rescue in behavior when compared to 5xFAD/Veh mice (**g**). Importantly, no groups in the 12-month timepoint significantly differed in total distance travelled during the trial (**h**). All analyses performed on this figure used a two-way ANOVA with Tukey’s multiple comparison test. Significance indicated as * *p* < 0.05; ** *p* < 0.01; *** *p* < 0.001

Having confirmed that HDs can pass the BBB and preferentially target PAMs in 5xFAD mice, we sought to determine the biological potential of these dendrimers through a proof of principle experiment. To that end, we used D-45113, a dendranib that inhibits CSF1R tyrosine kinase (Fig. 2a) which has a Kd of 0.04nM against CSF1R (Fig. 2b). D-45113 or HD (vehicle group) was administered to 4- and 11-month-old 5xFAD and WT mice (IP) twice weekly for 4 weeks (Fig. 2c), giving the following 4 groups: WT/Veh, WT/D-45113, 5xFAD/Veh, and 5xFAD/D-45113 (Fig. 2d). Mice were assessed with the elevated plus maze (EPM) task 24 h after the last injection and subsequently perfused 24 h later at which point, they were analyzed via immunohistochemistry (IHC), protein, and RNA analyses.

Anxiety behavior in mice was evaluated using the EPM as 5xFAD mice show robust impairments in this task. At 5 months of age, there are no apparent changes in behavior, as performance was similar across all groups regardless of genotype or treatment, as evidenced by their comparable time spent in the open arm of the maze (Fig. 2e). However, the distance travelled by 5xFAD/Veh mice is lower than that of both WT/D-45113 and 5xFAD/D-45113 mice, indicating that D-45113 may increase hyperexcitability in 5-month-old mice (Fig. 2f). At 12 months of age, 5xFAD/Veh and 5xFAD/D-45113 mice spend significantly more time in the open arm of the EPM compared to WT/Veh and WT/D-45113 mice (Fig. 2g), as expected [14]. Strikingly, time spent in the open arm is rescued in 5xFAD/D-45113 mice compared to 5xFAD/Veh mice, suggesting that D-45113 injection may improve behavioral outcomes in these mice (Fig. 2g). Notably, there is no significant difference in total distance travelled between any of the four groups at 12 months of age (Fig. 2h).

**Fig. 3:**
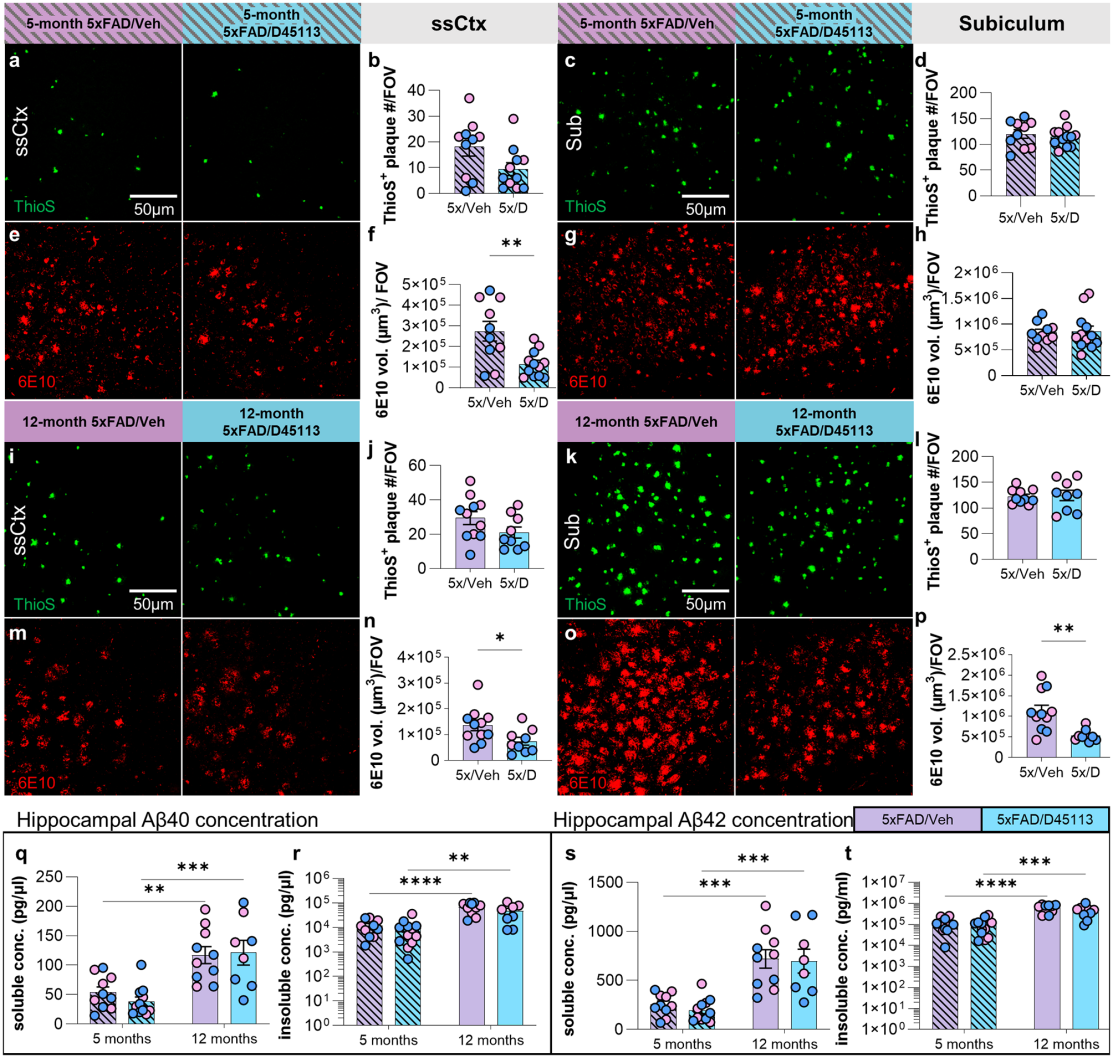
D-45113 treatment reduces Aβ plaque volume but not soluble and insoluble Aβ levels in the brains of 5xFAD mice. Representative 20x images of Thioflavin S (ThioS) staining in the somatosensory cortex (**a**) and subiculum (**c**) in 5-month 5xFAD groups, showing a nearly significant decrease in in ThioS^+^ dense-core plaques in the somatosensory cortex (**b**), but no change in the subiculum (**d**). 20x images of 6E10 staining for Aβ in the somatosensory cortex (**e**) and subiculum (**g**) in 5-month 5xFAD/Veh and 5xFAD/D-45113 groups reveal a significant reduction in 6E10 in the somatosensory cortex (**f**) of 5xFAD/D-45113 mice, but no change in the subiculum (**h**). Representative 20x images of ThioS staining in 12-month-old 5xFAD/veh and 5xFAD/D-45113 groups in the somatosensory cortex (**i**) and subiculum (**k**) and corresponding analysis shows a trending reduction in ThioS^+^ plaques in the somatosensory cortex (**j**), but not subiculum (**l**). 20x images of 6E10 staining in the somatosensory cortex **(m)** and subiculum (**o**) show striking reductions in 6E10 total volume in 5xFAD/D-45113 mice compared to 5xFAD/Veh controls in both brain regions (**n**, **p**). Hippocampal Aβ40 concentration measured via MSD assay shows an increase in soluble (**q**) and insoluble (**r**) Aβ40 levels in 12-month-old 5xFAD groups compared to 5-month-old 5xFAD groups, but no effects due to D-45113 treatment are observed. Similarly, soluble (**s**) and insoluble (**t**) hippocampal Aβ42 concentrations increase with age, but no changes are seen due to D-45113 treatment. Statistical analysis for (b, d, f, h, j, l, n, p) used a two-tailed t-test; (**q**-**t**) used a two-way ANOVA with Tukey’s multiple comparison test. Significance indicated as * *p* < 0.05; ** *p* < 0.01; *** *p* < 0.001

To investigate whether D-45113 treatment modulates pathological changes in the brains of 5xFAD mice, we performed IHC using Thio-S, an amyloid-specific dye for dense core plaques, and 6E10, an antibody against Aβ and APP. At 5 months of age in the somatosensory cortex, 5xFAD/D-45113 mice exhibit a trending reduction in the number of Thio-S dense core plaques (*p* = 0.057; Fig. 3a, b) and a significant reduction in extracellular 6E10 staining (*p* = 0.0034; Fig. 3e, f) compared to 5xFAD/Veh mice. However, in the plaque-heavy subiculum, no significant differences are observed between the two 5xFAD groups in the number of ThioS + plaques (Fig. 3c, d) or 6E10 staining (Fig. 3g, h). At the 12-month time point, a trending reduction in ThioS + plaque number (*p* = 0.11; Fig. 3i, j) and a significant reduction in 6E10 staining (*p* = 0.032; Fig. 3m, n) are observed in the 5xFAD/D-45113 group compared to the 5xFAD/Veh group in the somatosensory cortex. In the subiculum, no differences are observed in the number of ThioS + dense core plaques (Fig. 3k, l); however, a significant reduction in extracellular 6E10 volume was observed in 5xFAD/D-45113 mice compared to 5xFAD/Veh controls (*p* = 0.0025; Fig. 3o, p).

We measured the levels of insoluble and soluble Aβ40 and − 42 in the hippocampal tissue from 5xFAD/D-45113 and 5xFAD/Veh brains. As expected, the amount of soluble and insoluble Aβ40 is significantly higher at the 12-month time point compared to the 5-month time point. However, D-45113 administration has no effect on the concentration of Aβ40 in the hippocampus (Fig. 3q, r). Similarly, an age-dependent increase in soluble and insoluble Aβ42 is observed, but no changes in concentration were observed due to D-45113 administration (Fig. 3s, t).

### D-45113 treatment reduces the number of microglia in aged 5xFAD mice

**Fig. 4:**
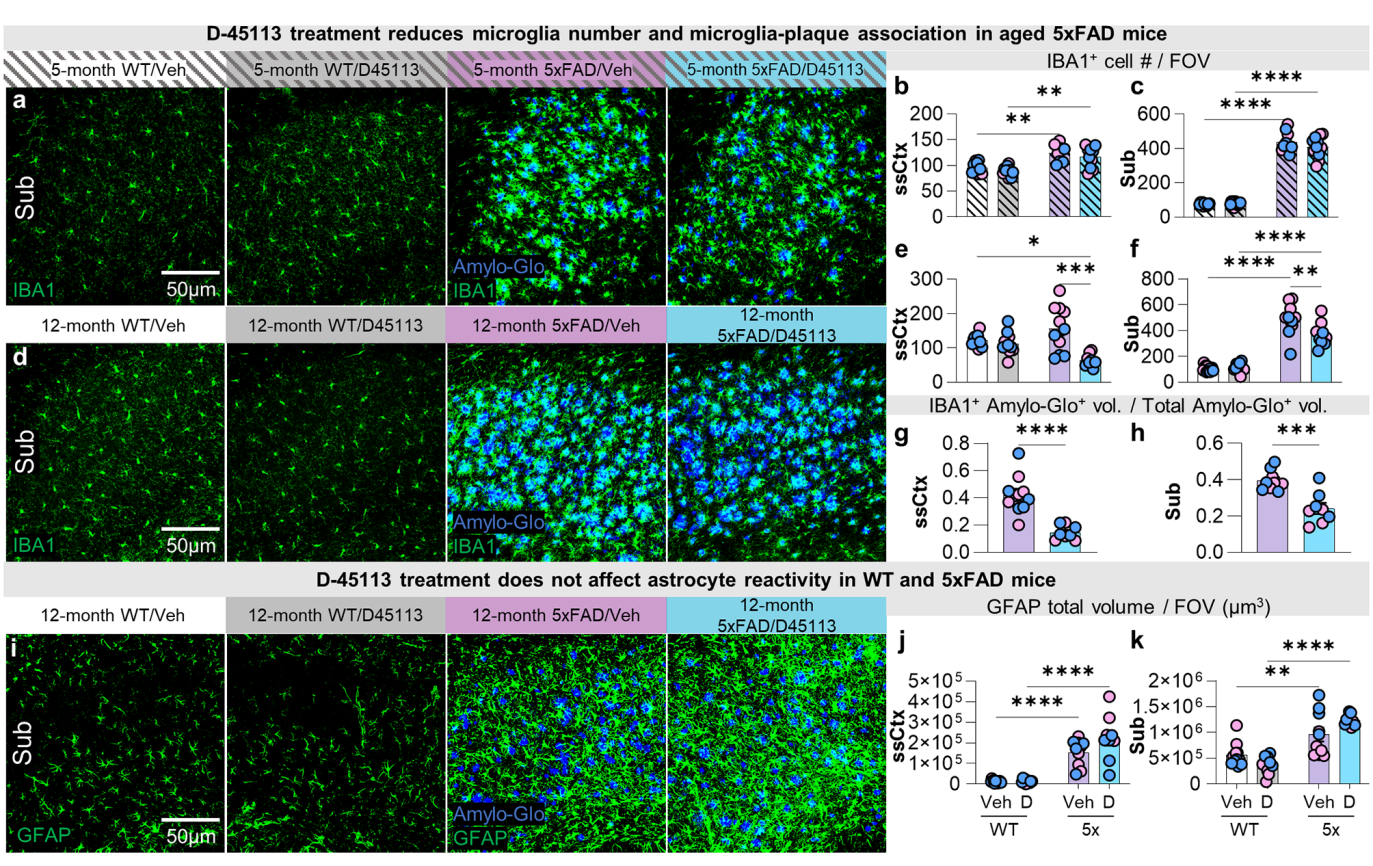
D-45113 treatment reduces the number of microglia but not GFAP + astrocytes in aged 5xFAD mice. Representative 20x images of the subiculum of 5-month-old WT/Veh, WT/D-45113, 5xFAD/Veh, and 5xFAD/D-45113 stained for IBA1 and Amylo-Glo (**a**). In both the somatosensory cortex (**b**) and subiculum (**c**), the number of IBA^+^ cells increase in the 5xFAD groups compared to the WT groups; however, no changes are observed due to dendrimer treatment. 20x images in the subiculum of 12-month-old groups stained for IBA1 (**d**) show a significant reduction in the number of IBA1^+^ cells in the 5xFAD/D-45113 group compared to the 5xFAD/Veh group in both the somatosensory cortex (**e**) and subiculum (**f**). At 12-months of age, 5xFAD/D-45113 mice show decreased colocalization between IBA^+^ microglia and plaques compared to 5XFAD/Veh mice in the somatosensory cortex (**g**) and subiculum (**h**). Representative 20x images of the subiculum of all four groups stained with reactive astrocyte marker, GFAP, and Amylo-Glo (**i**). In the somatosensory cortex (**j**) and subiculum (**k**) there is more GFAP volume in the 5xFAD groups compared to the WT groups, however, no changes occur with D-45113 treatment. Statistical analysis for (**g**, **h**) used a two-tailed t-test; (**b**, **c**, **e**, **f**, **j**, **k**, **m**, **n**) used a two-way ANOVA with Tukey’s multiple comparison test. Significance indicated as * *p* < 0.05; ** *p* < 0.01; *** *p* < 0.001

Having shown that HDs selectively target PAMs, we next investigated the effect of D-45113 treatment on microglia. We performed IHC on brain sections from 5- and 12-month-old WT/Veh, WT/D-45113, 5xFAD/Veh, and 5xFAD/D-45113 mice with the microglial marker, IBA1, and the dense core plaque stain, Amylo-Glo (Fig. 4a, d). At 5 months of age, we observe an increase in the number of IBA1 + microglia in both 5xFAD groups compared to the WT groups, as expected. However, no differences are observed due to D-45113 treatment in either the WT or 5xFAD mice at 5 months of age (Fig. 4b, c). Exploring the 12-month time-point, significant reductions in IBA1 + microglia cell number are induced by D-45113 treatment in both brain regions in 5xFAD mice, but not WT mice (Fig. 4e, f). Along with reductions in microglia number, microglia-plaque association is lowered in 5xFAD/D-45113 mice compared to 5xFAD/Veh mice in the subiculum and somatosensory cortex (Fig. 4g, h), indicating that D-45113 is able to modulate microglial activity.

We next investigated whether D-45113 treatment affected astrocytes, as microglial activation has previously been shown to induce astrogliosis [38]. We stained 12-month-old WT/Veh, WT/D-45113, 5xFAD/Veh, and 5xFAD/D-45113 mouse brain slices with the reactive astrocyte marker GFAP and Amylo-Glo (Fig. 4i) and measured GFAP + staining in the somatosensory cortex and subiculum. In both regions, we observe an increase in the GFAP + signal in both 5xFAD groups compared to both WT groups, consistent with previous literature [14]. However, no changes are observed due to D-45113 administration (Fig. 4j, k).

### D-45113 treatment in aged mice reduces microglial and inflammatory gene expression in 5xFAD mice

**Fig. 5:**
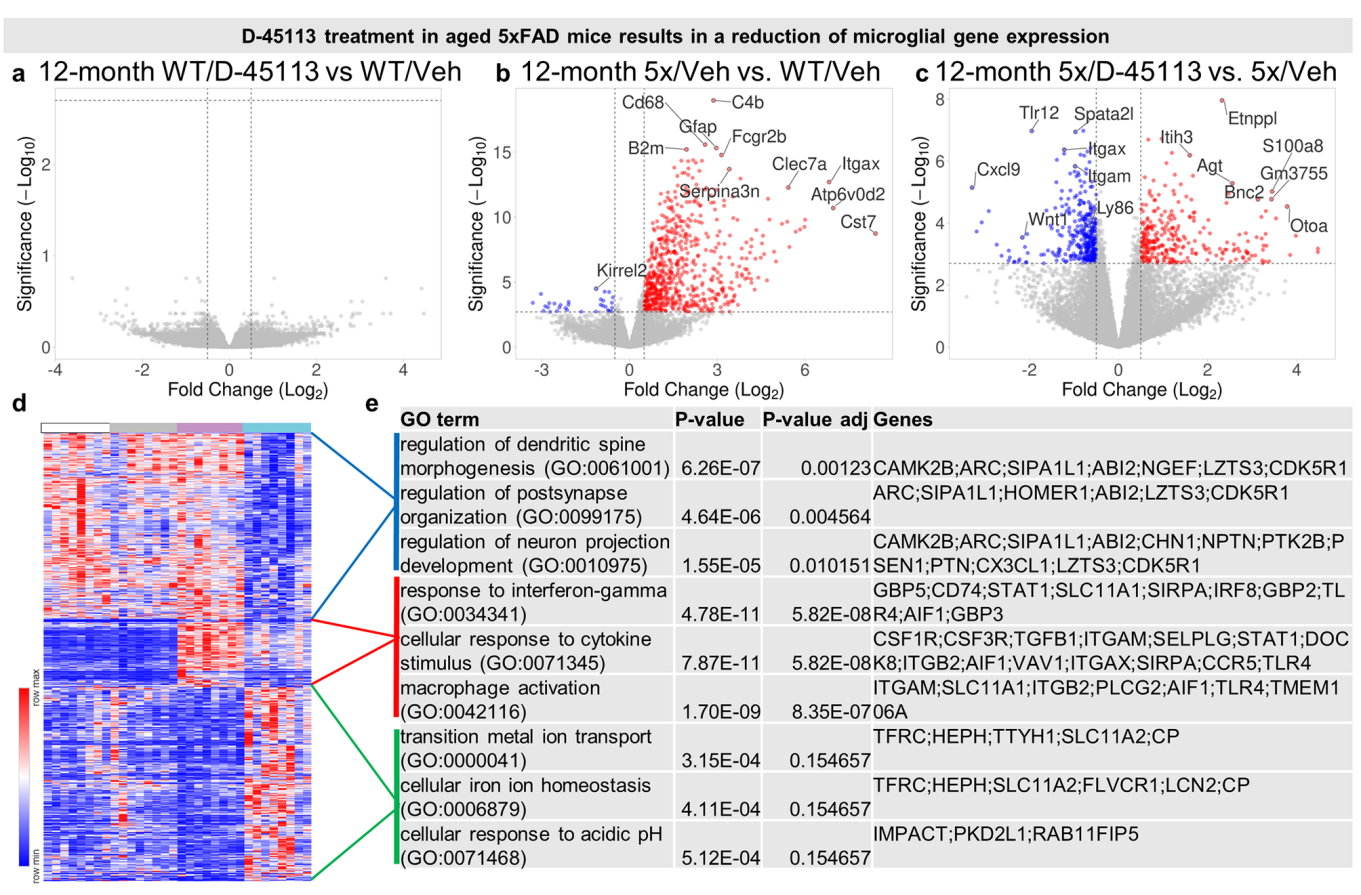
Bulk RNA-seq inflammatory gene expression is downregulated in 5xFAD mice treated with D-45113. Volcano plot looking at differentially expressed genes (DEGs) between WT/D-45113 and WT/Veh groups show no changes in gene expression between the groups (**a**). Volcano plot examining the DEGs between 12-month-old 5xFAD/Veh mice compared to WT/Veh mice reveals upregulation of classical AD inflammatory genes (*Clec7a, Itgax, Cst7*, etc.) (**b**). Volcano plot examining the DEGs between 12-month-old 5xFAD/D-45113 mice and 5xFAD/Veh mice reveals downregulation of microglial and inflammatory genes (*Itgax, Itgam, Cxcl9*, etc.) (**c**). Heatmap of individual FPKM values of the DEGs between the 12-month-old 5xFAD/D-45113 group and 5xFAD/Veh group for all mice analyzed via bulk RNA-seq (**D**). Gene ontology analysis on three distinct populations of DEGs (**e**)

To confirm the IHC changes due to D-45113 treatment, we performed bulk RNA sequencing (RNA-seq) on microdissected hippocampi from WT/Veh, WT/D-45113, 5xFAD/Veh, and 5xFAD/D-45113 mice. We first compared differentially expressed genes (DEGs) between WT/D-45133 and WT/Veh and found no significantly changed genes between the two groups, indicating that D-45113 does not have an effect on hippocampal gene expression in the WT mice (Fig. 5a). Consistent with literature [14], the expression of inflammatory genes is higher in 5xFAD/Veh mice compared to WT/Veh, with genes such as *Cst7*, *Itgax*, and *Clec7a* being highly upregulated (Fig. 5b). In contrast, 5xFAD/D-45113 mice have robust gene expression changes compared to 5xFAD/Veh mice, with a marked downregulation of genes associated with microglia and inflammation such as *Itgam*, *Itgax*, and *Cxcl9* (Fig. 5c). We generated a clustered heatmap from the fragments per kilobase of exon per million mapped fragments (FPKM) values for the 777 DEGs identified between 5xFAD/D-45113 and 5xFAD/Veh mice (Fig. 5d) and identified three clusters: genes that are downregulated with D-45113 treatment, genes that are upregulated in 5xFAD/Veh mice but rescued in 5xFAD/D-45113 mice, and genes that are upregulated with D-45113 treatment. We performed pathway analysis on the three clusters of genes through gene ontology (GO) analysis and listed the GO terms, P-value, adjusted P-value, and genes associated with the term in a table (Fig. 5e). The GO terms associated with the group of genes that are downregulated with D-45113 treatment are generally linked to synapses and neurons, with the top GO term being related to the regulation of dendritic spine morphogenesis. In the second group of genes, which are upregulated in 5xFAD/Veh mice but downregulated in 5xFAD/D-45113, the top GO terms are all linked to inflammation, with response to interferon-gamma being the top GO term. Finally, the third group, which contains genes that are upregulated with dendrimer administration, has GO terms linked to ion transport and homeostasis, but the adjusted P-values of the top GO terms are not significant. Overall, it appears that D-45113 treatment may impact inflammatory and dendritic and neuronal gene expression in the hippocampi of these mice.

### Axonal and neuritic damage are not rescued with D-45113 treatment

**Fig. 6:**
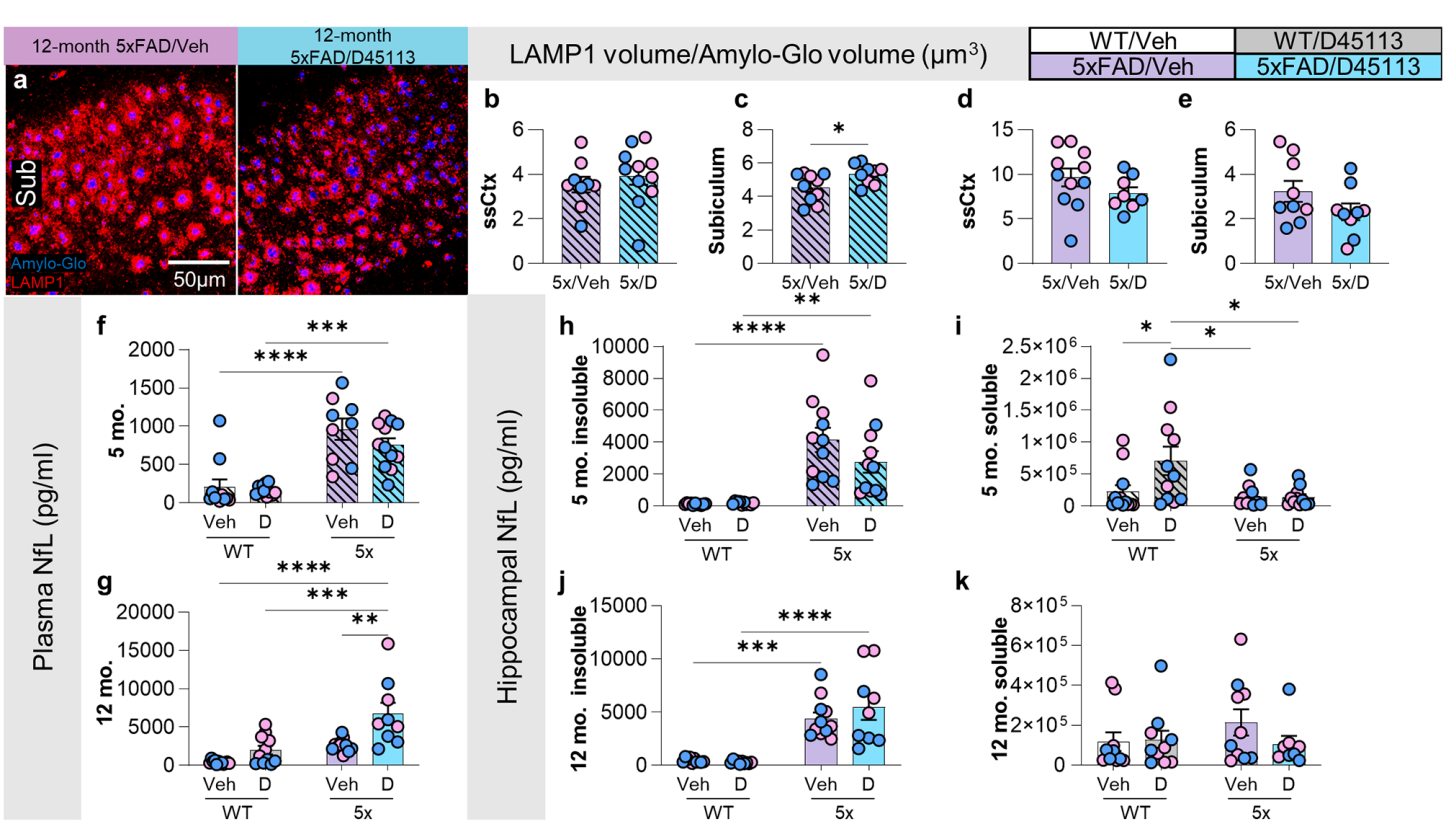
Axonal damage in 5xFAD mice is not rescued with D-45113 treatment. Representative 20x images of the subiculum of 12-month-old 5xFAD/Veh and 5xFAD/D-45113 mice stained with a marker for dystrophic neurites, LAMP1, and Amylo-Glo (**a**). There are no differences at 4-months of age in the somatosensory cortex between 5xFAD/Veh and 5xFAD/D-45113 mice in LAMP1 volume/Amylo-Glo volume (**b**), however, in the subiculum, 5xFAD/D-45113 show a higher level of dystrophic neurites (**c**). At 12-months of age, however, there are no differences in LAMP1 volume/Amylo-Glo volume levels in the somatosensory cortex (**d**) and the subiculum (**e**). Plasma neurofilament light chain (NfL) levels, which is a peripheral marker for axonal damage, increase in 5-month-old 5xFAD groups compared to WT groups but not with D-45113 treatment (**f**). At 12-months of age, D-45113 treatment in 5xFAD mice exacerbates plasma NfL levels (**g**). MSD assays measuring soluble and insoluble hippocampal NfL reveal an increase in insoluble NfL in 5xFAD groups compared to WT groups but no difference due to D-45113 treatment (**h**). In the soluble fraction, there is an increase in NfL in the WT/D-45113 group compared to the other three groups (**i**). At 12-months of age, both 5xFAD groups have higher hippocampal insoluble NfL levels compared to WT groups, but no changes are observed due to D-45113 treatment (**j**). No changes are observed in soluble NfL at 12-months of age are observed (**k**). Statistical analysis for (**b**-**e**) used a two-tailed t-test; (**f**-**k**) used a two-way ANOVA with Tukey’s multiple comparison test. Significance indicated as * *p* < 0.05; ** *p* < 0.01; *** *p* < 0.001

To explore synaptic and neuronal network differences between 5xFAD/Veh and 5xFAD/D-45113 mice, we stained 5- and 12-month-old 5xFAD/Veh and 5xFAD/D-45113 mouse brain tissue with the neuritic dystrophy marker, LAMP1, and Amylo-Glo (Fig. 6a). At 5-months of age in the somatosensory cortex, there is no significant difference between the 5xFAD/Veh and 5xFAD/D-45113 groups (Fig. 6b). However, in the subiculum, 5xFAD/D-45113 mice have higher LAMP1 staining adjusted for plaque load than 5xFAD/Veh mice (Fig. 6c). At 12-months of age, there are no differences in LAMP1 staining adjusted for plaque load in the somatosensory cortex (Fig. 6d) and subiculum (Fig. 6e). Neurofilament light chain (NfL) found in blood plasma has emerged as a potential biomarker for AD, with higher levels of NfL associated with higher axonal damage [35]. We measured plasma NfL levels of 5- and 12-month 5xFAD/Veh and 5xFAD/D-45113 mice. At the 5-month timepoint, there is an elevation in plasma NfL in both 5xFAD groups compared to WT groups but no differences with D-45113 treatment (Fig. 6f). At 12-months of age, 5xFAD/D-45113 mice have higher plasma NfL levels than 5xFAD/Veh mice (Fig. 6g). Measurements of NfL in the insoluble brain fraction reveal increases in 5-month-old 5xFAD groups compared to WT, but no changes are observed due to D-45113 treatment (Fig. 6h). In the soluble fraction, NfL in the WT/D-45113 group is elevated compared to the other three groups (Fig. 6i). Similar to the 5-month data, the 12-month timepoint shows an increase in insoluble hippocampal NfL in 5xFAD groups and no differences with D-45113 treatment (Fig. 6j), however, no changes in the soluble fraction are observed (Fig. 6k).

## Discussion

Dendrimers are dynamic nanomolecules which have been utilized for drug delivery in cancer and in the brain when the BBB has been severely compromised [56]. Factors determining dendrimer biodistribution and toxicity are chemical composition, architecture, size, and surface properties. Traditional PAMAM dendrimers have trouble passing intact or slightly impaired BBB [74]. Currently, there exist few ways to deliver therapeutics past the BBB and into the brain. One approach is through cerebrospinal fluid (CSF), intracerebral, and intracerebroventricular injection, however, while this is an efficient means to get therapeutics in the brain, these injections are very invasive procedures [11, 53]. Other non-invasive delivery methods such as nasal drug administration, exosome delivery, and nanoparticle delivery represent promising avenues for drug delivery to the brain, but these methods have their own caveats as well such as toxicity problems, dosing limitations, and drug conjugation problems [11, 21, 52, 57]. Previous research has shown that HDs can bypass a partially impaired blood-brain barrier and be phagocytosed by activated microglia and macrophages [44, 45, 47–49, 66]. However, to our knowledge, this is the first study to assess the ability of HDs to target and treat PAMs specifically in the context of AD. With this in mind, we aimed to address two main objectives: first, to investigate whether HDs can selectively target PAMs in the brains of 5xFAD mice; and second, to evaluate the potential of these HDs for biological modulation of PAMs in the brain.

To clarify the precise role of microglia in AD pathogenesis, it is critical to target specific subsets of microglia, particularly PAMs. Among the key regulators of microglial-plaque association in AD, the most extensively studied is triggering receptor expressed on myeloid cells 2 (TREM2). Previous research that involved knocking out, knocking down, or overexpressing TREM2 or its downstream effectors has emphasized the crucial role of TREM2 in promoting microglial association with plaques [17, 18, 26, 27, 33, 34, 54, 68]. Nevertheless, the impact of this association on the brain remains uncertain. This may be due to the fact that the vast majority of these studies have targeted all cells in the brain and periphery starting *in utero* in mouse models. In humans, TREM2 is essential for maintaining normal brain homeostasis [15, 23, 46, 65], and *TREM2* mutations resulting in loss of function are associated with a distinct neurodegenerative condition, Nasu-Hakola disease [72]. This represents a confound in the current literature and highlights the importance of developing therapies which target subsets of diverse cells with temporal specificity. HDs, which target only the most phagocytic microglia in AD, appear to be very promising in this regard. HDs have the ability to therapeutically modulate PAM in regions of inflammation while sparing other cell types of potential off-target effects. Furthermore, AD progresses at different rates in different brain regions, resulting in varying microglial responses throughout the disease’s course. These dendrimers may be beneficial in that they may target microglia only when needed in disease.

Here, we show proof of principle that HDs are specifically internalized by PAMs and can have a biological effect when conjugated to a CSF1R inhibitor (D-45113) in a mouse model of AD. While we find that dendrimer is only colocalizes with microglia in the brain, it is possible that other cell types may take up levels of dendrimers that are undetectable via IHC. Also worth noting is the fact that PAMs are more resistant to CSF1R inhibition-mediated depletion compared to NPAMs in 5xFAD mice and a mouse A/T/N model [39, 62], perhaps indicating that CSF1R may not be the ideal target to robustly modulate PAMs. Regardless, D-45113 administration in 5xFAD mice reduces microglia number, similar to previous studies which pharmacologically inhibit CSF1R [10, 12, 61]. Interestingly, with reductions in microglia number in 5xFAD mice treated with dendrimer, there is also a reduction in diffuse plaque volume (6E10); however, no difference in dense-core plaque volume (ThioS) is observed. This could in-part be due to the timing of treatment, as dense-core plaques are present in 5xFAD mice as early as 2 months of age [50]. Additionally, D-45113 treatment results in less microglia-plaque interaction and an increase in plasma NfL levels in 5xFAD mice. This falls in line with previous studies suggesting that microglia-plaque interaction is beneficial in limiting the amount of damage caused by Aβ plaques [13, 18, 62, 69, 70, 73]. These effects are much more prominent in older mice, as evidenced by behavioral, plaque, microglial, and RNA differences observed in 12-month-old 5xFAD mice treated with D-45113. Previous data from our lab indicate that PAMs show much higher levels of DAM marker CD11c (ITGAX) at 12- versus 4-months of age [67], perhaps suggesting that microglia in our older cohort of mice may be more prone to dendrimer uptake. Surprisingly, while treatment with D-45113 led to a rescue in EPM behavior and Aβ levels, and a lowering of inflammatory gene expression, there is no rescue in dystrophic neurite or NfL levels. A few reasons we may not see a rescue include: (1) We treated mice that are mid-late stage in disease pathogenesis and perhaps treating earlier and for longer than 28 days will lead to a rescue; (2) The partial reduction of PAMs is not sufficient to rescue the damage apparent in 5xFAD mice; and (3) D-45113 treatment may have independent effects on the brain of these mice. Our findings suggest that while D-45133 treatment may have therapeutic benefits, further investigation is needed to determine the mechanisms behind synaptic damage and rescue with treatment.

Here, we have shown that HDs have the capacity to target and treat PAMs with temporal precision through systemic administration. We were also able to show proof of principle that D-45113, a dendranib that inhibits the CSF1R has biological activity in the AD mouse brain, specifically in PAMs. Previous studies have shown successful conjugation of dendrimers with siRNAs, antisense oligonucleotides, and other commercially available drugs [7, 11], making these tools essential for delivering therapeutics across the BBB and directly to PAMs. Microglia have been increasingly implicated in tau hyperphosphorylation [5, 18, 34, 36, 37, 58], and future studies employing dendrimers in plaque + tau mouse models will be crucial to understand the interaction between microglia and the two primary histopathological hallmarks of AD. Overall, however, these results demonstrate that systemically administered HD’s can be conjugated to effector molecules and enact a biological effect on their target microglial population.

## Conclusions

Our results indicate that hydroxyl dendrimers (HDs) can cross a slightly impaired BBB and preferentially target PAMs in the brains of 5xFAD mice while leaving other cell types unaffected. Additionally, we show proof of principle that HDs conjugated to a CSF1R inhibitor (D-45113) can have effects on AD plaque pathology microglial number, plaque association, and transcription in 5xFAD mice. Ultimately, we show that HDs conjugated to effector molecules can have modulating effects on PAMs and further studies utilizing HDs should be undertaken to therapeutically target the PAM cell population.

## Data Availability

RNA sequencing data is available through GEO servers Accession GSE266470.

## Abbreviations

AD: Alzheimer’s Disease
Aβ: Amyloid Beta
BBB: Blood brain barrier
DAM: Disease associated microglia
GFAP: Glial fibrillary acidic protein
IBA1: Ionized calcium binding adaptor molecule 1
OLIG2: Oligodendrocyte transcription factor 2
PAM: Plaque associated microglia
ssCtx: Somatosensory cortex
Sub: Subiculum
TREM2: Triggering receptor expressed on myeloid cells 2
